# Protective Antibodies Against Influenza Proteins

**DOI:** 10.3389/fimmu.2019.01677

**Published:** 2019-07-18

**Authors:** Herbey O. Padilla-Quirarte, Delia V. Lopez-Guerrero, Lourdes Gutierrez-Xicotencatl, Fernando Esquivel-Guadarrama

**Affiliations:** ^1^LIV, Facultad de Medicina, Universidad Autonoma del Estado de Morelos, Cuernavaca, Mexico; ^2^Instituto de Biotecnologia, Universidad Nacional Autonoma de Mexico, Cuernavaca, Mexico; ^3^Facultad de Nutricion, Universidad Autonoma del Estado de Morelos, Cuernavaca, Mexico; ^4^Centro de Investigaciones Sobre Enfermedades Infecciosas, Instituto Nacional de Salud Publica, Cuernavaca, Mexico

**Keywords:** influenza A virus, neutralizing antibodies, non-neutralizing antibidies, influenza proteins, protective antibodies

## Abstract

The influenza A virus infection continues to be a threat to the human population. The seasonal variation of the virus and the likelihood of periodical pandemics caused by completely new virus strains make it difficult to produce vaccines that efficiently protect against this infection. Antibodies (Abs) are very important in preventing the infection and in blocking virus propagation once the infection has taken place. However, the precise protection mechanism provided by these Abs still needs to be established. Furthermore, most research has focused on Abs directed to the globular head domain of hemagglutinin (HA). However, other domains of HA (like the stem) and other proteins are also able to elicit protective Ab responses. In this article, we review the current knowledge about the role of both neutralizing and non-neutralizing anti-influenza proteins Abs that play a protective role during infection or vaccination.

## Introduction

The influenza proteins are recognized as foreign by the immune system, and antibodies (Abs) against them are produced during vaccination or after a natural infection. The Ab response can be *neutralizing* or *non-neutralizing*. *Neutralization* refers to the reduction of viral infectivity exerted by an Ab when binding to a virus. *Neutralizing Abs* inhibit virion cell entry because their epitopes are located near the receptor-binding site (RBS) on the globular head of HA. They can also interfere with the conformational changes necessary to expose the fusion peptide on HA (anti-stalk Abs). Despite the fact that neutralizing Abs are protective, the term neutralization has been often misused as a synonym of protection. Actually, these terms point to very different processes: whereas neutralizing Abs are defined by *in vitro* assays (e.g., hemagglutination inhibition and microneutralization assays), the term protection is associated to the reduction of morbidity and mortality *in vivo*. In this context, a minor fraction of *non-neutralizing Abs* that are generated upon the recognition of other viral epitopes can also be protective by other mechanisms, such as those that do not involve interfering the virus-cellular receptor interaction like increasing phagocytosis, activating complement or promoting antibody-dependent cellular cytotoxicity (ADCC) (1).

### Influenza Virus

The Influenza A Virus (IAV) is a negative-sense single-stranded RNA virus of the *Orthomyxoviridae* family. The virion contains eight gene segments, encoding for at least 11 viral proteins. These gene segments are associated to the nucleoprotein (NP) and the polymerases (PB1, PB2, and PA), which form the ribonucleoprotein (vRNP) complex. The vRNP complex is surrounded by matrix protein 1 (M1), which forms the core of the virion. This structure is covered by a lipidic membrane acquired from the host cell. This membrane contains the glycoproteins HA and neuraminidase (NA), which jointly represent over ninety percent of the protein present in the membrane. Furthermore, matrix protein 2 (M2) forms a homotetrameric structure that crosses the viral lipidic membrane and functions as a pH-dependent ion channel (2). Each virion only contains approximately 20-60 M2-channels. Finally, few molecules of the nuclear export protein (NEP, formerly named NS2) are associated with M1 within the virion (3).

The IAV infects human epithelial cells in the respiratory tract by binding HA to the sialic acid residues present on their surface; this is followed by virus internalization through receptor-mediated endocytosis. Upon endosome acidification, HA undergoes conformational changes that allow it to expose the fusion peptide that promotes viral-endosomal membrane fusion. On the other hand, the IAV core is also acidified by the entry of protons through the M2-ion channel. Both processes allow the vRNPs to be released into the cytoplasm, from where they are transported to the nucleus via nuclear localization signals (NLS) present in all vRNPs. Once in the nucleus, positive sense RNA is transcribed into mRNAs and replicated to produce a full-length complementary replicative intermediate (cRNA) by the viral RNA-dependent RNA polymerase. Afterwards, the mRNAs exit the nucleus to be translated by ribosomes, and the cRNA will serve as template to produce viral RNA (vRNA). Newly synthesized viral proteins come back to nucleus to assemble vRNPs, which, assisted by the NEP protein, will be exported to the cytoplasm where they are now ready for the packaging process in the cell membrane. The budding process of the newly assembled virions is largely facilitated by the M1 protein that recruits the necessary viral and host cell components. Finally, the NA promotes the viral exit process by pruning the interactions between sialic acid and the newly formed virions (2, 4).

Other non-structural (NS) proteins are produced during the IAV infection cycle. They play major roles in modulating the immune system to facilitate the infection. NS1 inhibits type I interferons by binding directly to RIG-I (retinoic-acid-inducible gene-I) and/or impeding its ubiquitination by interacting with the E3 ligase TRIM25 (tripartite motif-containing protein 25) (5, 6). PB1-F2 protein has been shown to have proapoptotic activity in epithelial and immune cells, such as macrophages (7). Finally, the PA-X protein degrades the host transcripts in the nucleus (6).

There are two major mechanisms of IAV evolution: *antigenic drift* and *antigenic shift*. The antigenic drift occurs frequently because of the poor fidelity of RNA polymerase that generates point mutations in the HA and the NA, that allow the virus to escape from neutralizing Abs. Eventually, these mutations are introduced into the circulating viral strains. This mechanism makes it necessary the annual revision of seasonal influenza vaccines. On the contrary, the antigenic shift occurs rarely, and consists in the generation of a completely new antigenic strain by the reassortment of gene segments during co-infections with human, avian and swine viruses. These evolutionary strategies are responsible for epidemics (antigenic drift) and pandemics (antigenic shift). The challenge posed by IAV is the generation of a “universal vaccine,” which could offer protection against any epidemic or pandemic strain (8).

### Influenza Virus Vaccines

Currently, there are three types of licensed human influenza vaccines: trivalent/quadrivalent inactivated vaccines (TIV/QIV) live attenuated vaccines (LAIV) and the recombinant vaccine Flublok. TIV/QIV are administered intramuscularly. They are non-adjuvanted inactivated vaccines composed of three or four circulating influenza virus strains (H1N1, H3N2, B; or two B strains for QIV). The strains are grown individually in embryonated chicken eggs and manipulated to harbor all internal genes from the A/PR/8/34 (H1N1) virus and two HA and NA genes corresponding to the circulating strains each year. There are three types of inactivated vaccines: whole virus vaccines, split virus vaccines, and subunit vaccines. In whole-virus vaccines, the allantoic fluid is harvested after the culture, and the virus is chemically inactivated with formalin or β-propiolactone. Split-vaccines add an extra step with detergent to make it less reactogenic by removing RNA. In subunit-vaccines, the HA of each virus is further purified. On the other hand, LAIV consist of cold-adapted virus, and they are administered intranasally. They do not replicate well in the lower respiratory tract, but they do in the nasal cavities. Flublok is a trivalent recombinant hemmaglutinin influenza vaccine, licensed by the FDA (US Food and Drug Administration) in 2013, that contains HA antigens derived from the three influenza virus strains recommended by the World Health Organization (WHO) annually (9).

For seasonal vaccines, the main mechanism of protection is the induction of neutralizing Abs specific for the globular domain of HA. This parameter can be measured by hemagglutination inhibition or neutralization assays, where a serum titer ≥40 is correlated to protection. Unfortunately, the effectivity of these vaccines depends on the accuracy of the virus strain selection process coordinated by the WHO. An inaccurate selection of strains may explain why the vaccines have shown low levels of protection in certain years (10).

### B-Cell Response Against the Influenza Virus: Learnings From the Mouse Model

The first Abs that participate in clearing an influenza infection are the so-called *natural Abs*, which are polyreactive Abs, mainly IgM, secreted by CD5^+^ B-1 cells present in pleural and peritoneal cavities. These Abs are continuously produced in the absence of infection, and they have low affinity for the antigens (11). The role of natural Abs in the influenza infection was addressed by Baumgarth et al., who showed that the passive transfer of naïve serum from wild-type to IgM KO (^−/−^) mice infected with influenza reduced the mortality in comparison to the controls (12). These Abs are present in airways in high levels, since they are transported to mucosal surfaces by poly-Ig-receptors located in the basolateral membrane of the alveolar epithelial cells (13). They could neutralize the IAV directly, or lyse cells by fixing complement (14). However, the levels of natural Abs are usually low, and most pathogens can overcome this barrier and establish an infection.

For an influenza-specific B-cell response to occur, the antigen must travel to secondary lymph organs (SLO) like draining lymph nodes and/or mucosa-associated lymphoid tissues (MALT). There, specific B cells encounter the antigen for the first time; then, they are differentiated to antibody-secreting cells (ASC) (15). Once in the LN or the spleen, the antigen may be captured, by two main types of cells; subcapsular sinus macrophages (SSM) and medullary dendritic cells (MDC), which capture the antigen mostly opsonized by complement components and thus, facilitate its encounter with the B cells. Then, the virus is transported and handed to the follicular dendritic cells (FDC), which retain it for continuous antigen presentation during long periods of time. These cells serve as major promotors of center germinal formation (15, 16).

Once the influenza antigens reach the draining lymph nodes, two types of B cell responses take place: the extrafollicular (EF) and the germinal center (GC) responses. Within the first days of infection (48–72 hpi), Abs are secreted by extrafollicular plasmablasts (short-lived antibody secreting cells). These early specific Abs play an important role in dealing with primary infections, because they contribute to ameliorate the disease outcome. This is mainly a T-dependent response, although some minor T-independent responses have been documented (17).

On the other hand, some virus-specific B cells migrate toward the marginal zone of the B follicles, where they interact with CD4^+^ T cells triggering a germinal center reaction (GCR) that leads, late during the infection, to the generation of long-lived plasma cells that maintain high levels of high-affinity Abs, which is the most desirable consequence of vaccination or infection, along with long-lived memory B cells (18). Briefly, in the GCR, follicular helper T cells (Tfh), which express CD40L and cytokines like IL-4, IFN-γ, and TGF-β, induce immunoglobulin class switching of activated B cells. Moreover, Tfh and B cells physically interact via ICOS/ICOSL, PD-1/PD-L1, CD28/B7 and other co-stimulating signals, leading to IL-21 secretion. Jointly, these signals promote somatic hypermutation and affinity maturation, resulting in influenza-specific high-affinity-ASCs. At the same time, during the GCR, some ASCs differentiate into memory B cells, which can be defined as cells that have undergone antigen-driven proliferation and have then become non-proliferating cells. They can be induced by re-exposure to the antigen and afterwards proliferate and secrete Abs. (15).

During subsequent IAV infections, GC response from GC-derived memory B cells dominate the response, however, the role of the EF B cells cannot be discarded since it has been shown in an antigen-specific experimental mouse model that the GC-derived memory B cells pool can respond as EF in a secondary response (19). In humans, high throughput sequencing of the B cell repertoire after infection or vaccination could help to understand the dynamic of EF and GC responses.

## Antibodies Against IAV External Proteins

### HA-Specific Antibodies

HA from influenza viruses is a spike-shaped protein that extends from the surface of the virus. The HA precursor (HA0) trimerizes in the ER and in the virion surface is processed by tissue trypsin generating two polypeptides: HA1 and HA2, which interact through disulfide bonds. HA1 comprises the globular region of the molecule (head), which contains the RBS, and the upper part of the stem region. HA2 covers the major part of the stem region, and it contains the fusion peptide. Currently, 18 different serological IAV HA subtypes have been described, and they have been divided into two phylogenetic groups: group 1 (including H1, H2, H5) and group 2 (including H3 and H7). In humans, H1 and H3 are the most frequent HAs present in circulating strains, and they are the main components of inactivated seasonal vaccines. However, some HAs from avian viruses such as H5 and H7 (e.g., H5N1 and H7N9) have crossed the interspecies barrier infecting humans and causing occasional outbreaks (20, 21).

#### Antibodies Against the Globular Domain of HA

##### Classical neutralizing antibodies: original antigenic sin

Most of the classical neutralizing Abs against influenza are directed to the conformational epitopes on HA, particularly the globular domain, which has been well-characterized as the immunodominant region of this protein. Since the early eighties, using monoclonal Abs (mAbs) as a tool, five non-overlapping sites (Sb, Sa, Ca1, Ca2 and Cb, or A-E sites) were identified as the major regions recognized by neutralizing Abs (22–25). Sites Sa and Sb are located at the top of the globular domain of HA, while Ca1, Ca2, and Cb are located at the bottom of the head (22).

Using a mouse model, it was shown by Angeletti et al., that there is a hierarchy among the five antigenic sites of the HA molecule of PR8 virus (H1N1), that depends on the immune response progress, the genetic background and the way in which the antigen is formulated and delivered: Cb-specific B-cells are predominant in the immediate response after infection, but they are substituted by Sb-specific B-cells at day 21. This hierarchy was not influenced by CD4^+^ T cells, and it may change with different administration routes and different strains of mice (26). Later, Liu et al., analyzed the hierarchy of immunodominance for the HA of a post-2009 influenza pandemic strain, A/Michigan/45/2015 (H1N1) in several species including humans: while no specific immunodominance pattern was found with guinea pigs, Sb and Ca-specific Abs dominated the immune response in mice and the site Sa was dominant in ferrets. For humans it was reported a completely different pattern in which Sa and Sb-specific Abs dominated the antibody response (27). Similarly, for H3 virus, Broecker et al. found that the B site (analog to sites Sa and Sb in H1 HA) in the HA protein of the H3N2 strain that circulated in the 2017–2108 season was immunodominant pre and post-vaccination in humans that received seasonal vaccine. The same pattern of immunodominance was found with mice, but unlike reported by Angeletti et al., it was independent of genetic background and immunization route (28, 29).

Abs elicited against the HA globular domain during infection or vaccination usually are strain-specific, and they will hardly neutralize subsequent influenza virus strains (homosubtypic protection). This is explained by the selective pressure exerted by the immune system, which leads to the rise of new strains (with minor amino acid substitutions in the five neutralizing sites of HA head) that can avoid previous Abs (escape mutants). This evolutionary mechanism (antigenic drift) makes it necessary the annual reformulation of seasonal influenza vaccines.

Only specific Abs for the head of HA efficiently prevent infection, by blocking the HA-mediated attachment to the cell surface (26). Anti-HA Abs can also have an effect on the activity of other influenza-related proteins. Several authors have found both in humans and mice that anti-HA Abs can also interfere with the activity of neuraminidase (NA) by blocking virus binding to the surface bound NA-substrate or by sterically inhibiting NA access to the substrates (30–32).

An important feature of the neutralizing anti-IAV Ab response, predominantly involving the globular domain of HA, is the phenomenon called *original antigenic sin* (OAS). The term was coined in 1960 by Thomas Francis to describe the fact that in humans, influenza virus infections in childhood leaves an immunological imprint that results in high Abs titers against the childhood encountered virus after being boosted by new drifted virus (33, 34). These Abs are mainly directed against the conserved epitopes present in the different virus strains. A possible explanation for this phenomenon, is that there is a competition between memory B cells specific for the first strains and naïve cells specific for the new strain, which need to meet more requirements for activation, such as higher antigen doses (35). Another interesting hypothesis to explain the OAS, is that the T regulatory cells induced by the first antigen reduce the amount of the second antigen available to activate naïve B cells (36).

An example of OAS was observed in the most recent pandemic caused by an IAV H1N1 in 2009 (pH1N1/2009). As previously stated, the HA head does not induce a high level of cross-reactivity. However, the frequency of severe disease among elderly people infected with the pandemic strain was lower than it was among younger individuals, suggesting preexisting immunity. In this regard, IAV HA is more closely related to the 1918 pandemic virus A/South Carolina/1/1918 (H1N1) than HAs from seasonal strains, and those individuals who more likely experienced pre-1957 H1N1 strains had higher titers of neutralizing Abs to the 2009 H1N1 strain (37, 38). An interesting fact was that the main antigenic determinants of these Abs were located on the Sa site of the globular domain of HA, shared between the 1918 and 2009 strains (39).

Despite the fact that the term OAS was proposed almost 60 years ago, it is still valid, and elucidating the role of this phenomenon in infection and vaccination processes continues to be relevant. In a recent study Lindermann and Hensley found by using serum passive-transfer experiments in a mouse model, that Abs with an OAS phenotype were effective in neutralizing antigenically different influenza virus strains *in vivo*, indicating that OAS-Abs are an important mechanism of protection in secondary immune responses (40). However, according to two other studies, this phenomenon seems to have no impact on the response to vaccination in humans (41, 42). Further studies are necessary to determine more precisely the role of OAS after infection or vaccination against IAV.

##### Anti-HA head broadly neutralizing antibodies

Despite the fact that most anti-HA head Abs are strain-specific, in 2009 the mAb S139/1 was isolated from a mouse immunized with an H3 virus. Surprisingly, this Ab neutralized multiple subtypes, including H1, H2 and H3 strains, and its epitope is located in the antigen site B near the RBS (43).

In the same way, the human mAb CH65 was isolated from an adult resident of the United States that had received the 2007 TIV. According to crystallographic studies with the A/H1N1/Solomon Islands/3/2006 strain, CH65 Ab mimics the physiologic interaction between sialic acid and HA, as this antibody binds directly to the sialic-acid pocket through its HCDR3. In this report, CH65 neutralized 30 out of 36 influenza H1N1 strains *in vitro* (44). Other receptor-binding site mAb (C05) was isolated by Ekiert et al. using phage antibody libraries from a human donor. The mAb C05 binds directly to RBS on HA using mainly its HCDR3 and with minor interaction through its HCDR1, and is capable to neutralize group 1 and group 2 influenza virus strains (45).

Furthermore, anti-head broadly neutralizing mAbs whose epitopes are farther from the RBS have been described. Ohshima et al. isolated mAbs F045-092 and F026-427 from human B lymphocytes. These mAbs showed activity against the H1N1, H3N2 and H5N1 viruses, and their epitopes were also found to be on the globular head of HA (46). D1-8 is a human mAb whose epitope is close to the D antigenic site, different from the RBS, and it is highly conserved among the H3N2 viruses. In mice, it has a better therapeutic effect than oseltamivir (47).

#### Antibodies Against the HA Stem

##### Anti-HA stem broadly neutralizing antibodies

In contrast to the globular domain of HA, the stem domain (or stalk domain) of HA is far less variable, and it has been shown to induce broadly neutralizing Abs (bnAbs). In 1993, Okuno et al. described for the first time a mAb (C179) specific for the HA stem region in mice. It had no hemagglutination inhibition activity (HAI), but it was capable of neutralizing H1 and H2 viruses (group 1) (48). Recently, a number of mAbs, which have displayed protective activity in mice and have a broad range of neutralization activity for group 1 (CR6261), group 2 (CR8020), and both groups of influenza viruses (FI6) (49–51), have been described in humans. Unfortunately, their epitopes are subdominant after infection or vaccination and, therefore, new strategies have been proposed to boost the generation of Abs against the stem domain.

Most human anti-stem Abs, particularly those against HAs from group 1 (e.g., CR6261 and F10), use the V_H1−69_ gene family. These broadly reactive Abs are characterized by a phenylalanine in position 54 at the HCDR2 region unique to the V_H1−69_ gene. This provides them with a unique ability to form hydrophobic interactions with the hydrophobic groove between HA1 and HA2, using only their heavy chains. Thus, they inhibit the conformational changes necessary for the fusion of viral-cell membranes (52–54).

Regarding group 2 Abs, prototype human mAb CR8020 binds a different epitope from that of CR6261/F10. Although the epitope is also on the HA stem, it is closer to the virus membrane. This antibody uses both heavy and light chains to make contact with its epitope, and the fusion peptide accounts for 50% of the Van der Waals forces involved in the Fab-HA binding (50).

The human mAb FI6 recognizes both groups of HAs, since it is able to bind to their fusion peptide. This mAb was isolated from human plasmablasts. Its heavy and light chains correspond to the V_H_3-30^*^18 and V_K_4-1^*^01 gene families, respectively. Although this mAbs binding site overlaps with that of mAb CR6261/ F10, FI6 makes contact only with the HCDR3 region, while CR6261/F10 encompasses all three HCDR regions (49). Recently, S9-1-10/5-1 was described as a human mAb that uses the gene V_H_4-59 family and displays specificity to both HA groups. Although it binds to the HA2 A-helix, apparently it does not inhibit the virus entry. Instead, it binds to HA on the surface of the infected cells, thus preventing the viral particle release (55).

Although the occurrence of anti-stem Abs is low after a seasonal infection or vaccination, several reports indicate that these Abs are boosted after sequential infections or immunizations with viruses containing different types of globular HA, but essentially the same stem HA (56). Recently, Nachbagauer et al. analyzed the cross-reactivity pattern of anti-HA Abs after an influenza infection in patients diagnosed with pH1N1/2009 or seasonal H3N2, and they found that a pH1N1 infection induces a broader response (against group 1 and group 2 HAs) than an H3N2 infection does. This can be explained because the 2009 pandemic strain had a novel HA head, compared with that of seasonal viruses, and thus, could boost the response against the HA stem region (57).

With respect to the 2009 pandemic vaccination, Cortina Ceballos et al. analyzed the B cell repertoire in individuals, with no previous exposure to pH1N1/2009, after they received the monovalent inactivated vaccine containing the pandemic strain (09 MIV). They reported heterosubtypic neutralizing seroconversion in 17% of the individuals. The phenomenon was associated to a clonal expansion of B cells that used the V_H1−69_ segment and to other cells involved in the generation of anti-stem Abs (58). In the same context, Li et al. analyzed B-cell responses from vaccine-induced plasmablasts in healthy adults after they had received 09 MIV. They observed high levels of cross-reactivity against the HA-stem domain. This cross-reactivity pattern occurred in the case of pandemic vaccination, and it was not seen with the seasonal TIV. Furthermore, they found that, just like seasonal vaccines (TIV), anti-stem Abs had arisen from preexisting memory B cells even before the emergence of the 2009 pandemic virus, which suggests that they were induced by previous strains (59).

Additionally, the repertoire of B cells from individuals vaccinated in consecutive years with the pandemic strain pH1N1/2009 was analyzed by Andrews et al. They showed that the individuals with low basal levels of Abs specific for this strain generated a broadly reactive response directed mainly against the HA stem. On the other hand, individuals with high levels of Abs before vaccination correlated with a dominant response against the HA head domain after immunization. The authors suggest that the repertoire of anti-stem B cell memory is preexistent and that the immunodominance of the HA globular domain prevails with the subsequent encounters with the influenza virus (60). This observation echoes the dilemma of producing a universal vaccine that promotes the generation of anti-stem Abs or rather using the Abs in passive immunization strategies in infected individuals, since it is possible that consecutive challenges with seasonal strains of influenza will move the balance in favor of anti-head Abs.

It is well-known that anti-stem Abs are less permissive to virus escape, but Choi et al. identified three escape mutants in virus strain A/Perth/16/2009 (H3N2) after it was co-cultured *in vitro* with the human mAb 39.29, which neutralizes all IAV subtypes. The authors described that mutant Gly387Lys totally eradicates the antibody binding, while mutants Asp391Tyr and Asp391Gly increase the ability of HA to fuse membranes with just a slight interference in binding at low pH (61).

### NA-Specific Antibodies

Neuraminidase is the second most abundant glycoprotein on the surface of the influenza virion. It is a homotetramer with a mushroom-like form, and it plays two major roles during the IAV infection: It promotes adhesion to the receptors on the epithelial cells because it degrades mucus, and it facilitates viral exit by breaking the interactions between sialic acid and the newborn virions. Neuraminidase inhibitors like oseltamivir act by inhibiting the last step and causing virus aggregation on the cell surface. Each NA monomer is composed of approximately 470 amino acids that form four domains: a short cytoplasmic N-terminal domain that is 100% homologous among influenza strains, a transmembrane hydrophobic domain, and a stem-shaped C-terminal domain of variable longitude, which ends in a globular domain where the enzymatic site is located (62).

Anti-NA Abs have historically been underestimated, due to the central role that HA has played in influenza research. However, for the last 50 years, important data have been gathered suggesting that anti-NA Abs can offer protection against the influenza infection. In 1968, Schulman et al. demonstrated that Abs against this protein are produced in mice after an IAV infection. The outcome of an infection in naïve mice improved when NA-immune serum was transferred to them (63). The same research group confirmed that anti-NA Abs were also present in humans after an influenza infection (64). Later, Murphy et al. investigated the role of anti-NA Abs in a clinical study carried out with volunteers, with low basal levels of anti-HA Abs and variable levels of anti-NA Abs. These subjects were infected with influenza virus A/NT/60/68 (H3N2), and the authors observed that the individuals who displayed minimal symptoms had higher levels of anti-NA Abs (65).

Recently, Chen et al. found in humans that seasonal vaccination induces a poor NA-specific B-cell response, whereas anti-NA B-cell responses after an IAV infection are similar (H1N1) or even higher (H3N2) when compared to HA-specific B-cell responses. The authors also found that anti-NA Abs were cross-reactive to NA proteins from most IAV strains and that they showed prophylactic and therapeutic potential when evaluated *in vivo* (66).

NA-specific Abs induce infection-permissive immunity by limiting the viral load through interference with the exit of the virions. In other words, they do not prevent infection, but they contribute to ameliorate the clinical symptoms of disease. Among these Abs, those that are directed to the enzymatic site have the highest activity of neuraminidase inhibition (NAI), because they apparently limit the access of natural substrate to the catalytic site (67). Furthermore, anti-NA Abs are able to exert immune pressure within the globular domain of NA by promoting escape mutants (antigenic drift), which is an indirect proof that they play a role in immunity against IAV (68).

In the case of anti-NA Abs, there is also evidence of original antigenic sin. As stated previously, during the last IAV pandemic (pH1N1/2009), there was a low incidence of illness among elderly people. This was attributed to their previous exposure to similar IAV strains during childhood, which induced a recall response to the conserved domains of HA present in the strains. Similarly, Marcelin et al. found that NAI Abs were present in the sera from older people, and seroconversion was only registered in the age group ≥ 70 years after TIV vaccination. This provides evidence that NA-specific B cells from past strains were activated by the pH1N1/2009 virus, and they contributed to the protection process (69). In the same way, Rajendran et al., found that anti-NA Abs levels are directly proportional to age, and their reactivity are highest against influenza virus strains that more likely circulated during their childhood [A/South Carolina/1/1918 (H1N1), and A/Singapore/1/1957 (H2N2) in elderly; A/USSR/92/1977 (H1N1) and A/Philippines/2/1982 (H3N2) in adults] (31).

A unique opportunity to elucidate the independent contribution of the anti-NA Abs to the protection process was the 1968 Hong Kong IAV pandemic, during which a new virus (H3N2) emerged. It had a new HA, while the NA remained the same as in the circulating seasonal strain. Thus, evidence pointed out that anti-NA Abs played a key role in reducing the severity of the disease (70).

It is well-known that the gold standard for evaluation of vaccine efficacy is the HAI titer, where a value ≥40 is taken as protective by the FDA in the United States (71). Nonetheless and despite the lack of data regarding the contribution of anti-NA Abs in protection, Memoly et al. studied in humans the role of the NAI titer levels in predicting protection against influenza. They found that the same value of NAI titers (≥40) correlated better with the prediction of protection, even at higher levels than the HAI titer, which is only associated to a reduction of virus shedding. High levels of NAI titers also correlated with the reduction of the viral load and the duration and severity of the infection, among other symptoms (72). Similarly, Couch et al., confirmed by multivariate analysis that anti-NA Abs titers in serum and nasal secretions are independent predictors of immunity and protection to influenza in samples taken pre and post pandemic of 2009 (73). These results suggest that in addition to HAI, also NAI titers can serve as predictors of protection.

Anti-NA Abs are also produced in response to the administration of seasonal vaccines. Recently, Monto et al. showed that 37 and 6% of human recipients of TIV and LAIV, respectively, had Abs with NAI activity, whereas the values of HAI for these same groups were 77 and 21.2%, respectively. They also reported that after the 2007–2008 influenza season, NAI levels in subjects with confirmed infection rose to 41% for TIV, 63% for LAIV, and 76 % for unvaccinated subjects, whereas HAI levels were 18%, 77% and 97%, respectively (74).

Regarding cross-protection of anti-NA Abs, mice vaccinated with different recombinant NA resulted in reduction of mortality against influenza virus challenges with heterologous (not heterosubtypic) strains. This protection was dependent of specific-NA Abs, as shown by passive transference experiments (75). Additionally, Sandbulte et al. found that anti-N1 Abs can protect mice from a lethal challenge with the avian H5N1 subtype when previously immunized with a DNA vaccine encoding for N1 from human virus A/New Caledonia/20/99 (H1N1). Furthermore, they showed that human Abs detected in 81.6% (31/38) of the subjects were capable of inhibiting NA activity against the avian strain, suggesting that the incorporation of NA to TIV vaccines or the natural infection could offer protection against new pandemic strains such as H5N1(76). In this respect, Gillim-Rose and Subbarao debated Sandbulte's hypothesis pointing out that these data are still insufficient to predict a protective heterologous response to H5N1 in the human population. In consequence, forthcoming studies should focus on the magnitude and biological advantage of cross-reactive N1 Abs before considering the inclusion of this IAV protein in a vaccine (77).

Up to now, discussion has focused on the possible incorporation of NA in an anti-influenza vaccine. However, more information is required to determine the amount of antigen, the serologic data of NAI titers, and the type of vaccine to achieve the best protective immune response in humans. In this regard, present vaccines are designed for the production of anti-HA Abs, while the NA content has not yet been standardized (78). Attenuated vaccines present the same concern as a natural infection, since they contain a considerable higher proportion of HA than of NA (5:1) in the virion, which leads to an antigenic competition, where the HA-specific B-cell response overcomes the NA-specific B cell response (68, 79, 80). However, this antigenic superiority of HA over NA in terms of antibody production observed both in the natural infection and with vaccination is lost when proteins are administered separately and in the same proportion (81). Altogether, NA is a promissory candidate for the design of better vaccines against IAV. However, it seems that current data on NA-immunity is still insufficient. In this regard, for a more specific review, a recent publication addressed thoroughly the major knowledge gaps, pointing out the actions that should be taken on this matter (82).

### Fc Receptors (FcR)-Mediated Effector Functions for HA- and NA-Antibodies

In addition to previously described mechanisms of protection for HA-Abs, indirect antiviral FcR-mediated effector functions like ADCC, antibody-dependent cellular phagocytosis (ADCP) and complement mediated cell-cytotoxicity (CDCC) have been described both in humans and mice (83–92). Also, ADCC has been described for NA-Abs (87). Although, these mechanisms of protection will not be addressed further in this article, these effector antiviral function of HA- and NA-Abs may have an important role on protection against IAV infection.

### Antibodies Against M2 Protein

Matrix protein 2 (M2) is the third most abundant protein on the IAV virion surface. It is a type III integral protein arranged as a homotetrameric channel linked by disulfide bonds, which function as proton selectors. They induce the acidification of the virions and consequently the dissociation of the vRNPs from matrix protein (M1) and their release into the cytoplasm during the entry phase of the IAV cycle. The M2 protein is 96 amino acids long, and it has three domains: a cytoplasmic C-terminal (54 aa), a transmembrane (19 aa), and a short and highly conserved N-terminal ectodomain (M2e, 23 aa). Antiviral drugs amantadine and rimantadine target M2, blocking the proton influx into the virion through an allosteric effect (93).

The density of M2 in the virion is low (approximately 60 molecules/virion) compared to the high concentration of HA or NA on the viral membrane. These major glycoproteins also exert an allosteric blockade of M2, which makes it difficult to be reached by B-cell receptors and thus, it generates minimal immunogenicity during a natural infection. However, the N-terminal ectodomain of M2 (M2e) has been targeted in the design of a “universal vaccine,” because it is highly conserved among influenza strains, and because the capacity of anti-M2e Abs to generate heterosubtypic protective responses has been observed in mice (94).

The immunogenicity of M2e was first reported in 1988 by Zebedee and Lamb. They described a mAb (14C2) that was produced in mice immunized with M2 protein plus adjuvant. This mAb recognized the ectodomain of the protein, and it was able to detect M2 on the virions, thus reducing viral growth. This was evidenced by the size reduction of lytic plaques when 14C2 was added to previously IAV-infected MDCK cells (95). Later, Treanor et al. proved that this antibody reduced lung viral titers when ascitic fluid was passively transferred to naïve mice that were afterwards challenged with IAV (96).

Abs against M2 are not neutralizing. Nonetheless, due to the high expression of M2 on the surface of infected cells, they can contribute to the protection process by promoting effector functions based on their Fc region. Lee *et al*. reported that anti-M2e Abs were not protective in Fc receptor common γ-chain deficient mice (FcRγ^−/−^) in comparison to the high protection observed among wild-type mice in passive transfer experiments (97). In this context, El Bakkouuri et al. reported in a mouse model that protection induced by these Abs depended on phagocytosis of infected cells by alveolar macrophages (AM) by engagement to the Fc receptors (FcγRIII for IgG1, and FcγRI and/or FcγRIV for IgG2a) present in these cells (98). Furthermore, NK cells can induce ADCC by binding to the Fc domain of anti-M2 Abs. Simhadri et al. showed that freshly isolated and cytokine-preactivated NK cells in presence of a human anti-M2 antibody (1-10 mAb) can exert ADCC and secrete cytokines (99). The role of CDCC in M2e immunity is controversial: Jegerlehner et al. reported that anti-M2e Abs do not eliminate infected cells by CDCC (100), whereas Wang et al., reported that complement is necessary for an anti-M2e mAb to control lung viral titers in challenged mice (101).

Several reports in mice have shown that M2e can induce an efficient heterosubtypic protection. Different approaches have been used to determine this, such as coupling M2e to carrier proteins —like the hepatitis B virus core protein (HBc) (102)— or to flagellin (103); conjugated to nanoparticles of gold (104); inserted in VLPs (105); as DNA vaccines (106), and others. Recently, the efficacy of Abs against HA (induced by TIV), against NA (recombinant N1 and N2) and against M2 (M2e5XVLP) was compared in mice. It was found that immune sera against NA and M2e were superior in terms of improving heterosubtypic protection and survival than anti-HA Abs induced by the split seasonal vaccine. Interestingly, the co-administration of NA and M2e5XVLP immune sera gave rise to a synergistic heterologous protection effect (107).

In general, the levels of M2-specific Abs in sera of IAV infected patients are low and non-durable (108, 109). However, one study has suggested that anti-M2 Abs may increase with age after a pandemic strain appears. It is explained that a recall humoral response to this protein could be boosted, since the presence of anti-M2 Abs after infection with the pH1N1/2009 strain was detected in nearly 50% of the samples tested, even before anti-HA Abs specific to this strain could be identified (109).

Moreover, anti-M2e Abs have shown to be protective in humans. In a controlled challenged study, the administration of a specific anti-M2e IgG mAb (TCN-032), showed a reduction of 35% of symptoms compared to group that received placebo, when challenged with influenza virus A/Wisconsin/67/2005 (H3N2) (110). Also, several phase I and II clinical trials of M2e-based vaccines have shown to be safe and immunogenic in humans (103, 111, 112), and recently a phase I clinical trial started to evaluate a hepatitis B core-M2e-based vaccine in Russia (NCT03789539) (113).

## Antibodies Against Internal Proteins

The IAV infection induces Abs against internal and non-structural proteins, such as NP, M1, PB1-F2 and others (87, 114–116). Nevertheless, the protective role of these Abs is still unknown, although few studies in mice have shown that at least the anti-NP Abs can weakly help to clear influenza infection (117, 118).

The aa sequence of NP is conserved up to 90%, among various strains of influenza and heterosubtypic immunity (HSI) induced by this protein has been fully demonstrated in the mouse model, a feature that had been totally attributed to T cells (119–121). However, Rangel-Moreno et al. reported that T cells are insufficient to achieve HSI, and they proposed that non-neutralizing Abs contribute to decrease the severity of the illness by lowering viral titers, decreasing weight loss, and promoting the recovery of mice by helping CD8 T cells to expand after the heterosubtypic challenge (122). Furthermore, Carragher et al. analyzed the role of anti-NP Abs on HSI by vaccinating mice in the absence of T cells with recombinant nucleoprotein (rNP). They found that HSI was still present. However, it was lost when the Abs were absent, and it was recovered by transfer of rNP-immune serum (118).

Previous studies have shown that NP can be expressed on the surface of influenza virus-infected cells (123–125), however evidence for Fc-mediated effector functions of anti-NP Abs is controversial. Regarding ADCC, despite Varderven *et al* reported that healthy individuals had anti-NP and anti-M1 Abs capable of activating NK cells through FCγRIII, these Abs had no killing activity on target cells *in vitro* (116). Contrarily, Jegaskanda et al. found in human sera higher titers of NP-specific ADCC-Abs reactive to avian influenza strain H7N9, as compared with HA- or NA-specific ADCC-Abs reactive to the same strain. In addition, these Abs correlated with ADCC-Abs reactive to NP in the seasonal influenza viruses (H1N1 and H3N2), suggesting that they could be induced by seasonal infections or by vaccination (87). Also, Bodewes et al., reported no complement-dependent cell cytotoxicity using a human mAb specific for NP *in vitro* (124), while Yewdell et al. found low CDCC activity with five different mouse NP-mAbs in complement-mediated ^51^Cr microcytotoxicity assays (123).

LaMere et al. described in mice that anti-NP IgG Abs also contribute to the protection against IAV in a mechanism dependent on CD8^+^ T cells and Fc receptors (117). This can be explained because anti-NP Abs can associate with viral proteins (probably from dying infected cells) forming immune complexes (IC), which are captured by dendritic cells via FcγR, and promoting a sustained antigen presentation to CD8 T cells. All of this contributes to memory development (126). In accordance with this, when aged mice with a depressed cytotoxic T lymphocyte (CTL) response received artificial IC consisting of a NP-specific mAb and the influenza virus, the CTL response was restored, along with an enhanced dendritic cell function and an increment of IFN-γ by CD4^+^ and CD8^+^ T cells (127).

Finally, other internal and non-structural proteins like PA-X and PB1-F2 have shown to induce Abs, even though their role in protection has not been determined. In 2012, protein PA-X was identified as a product of the ribosomal frameshifting of IAV segment 3, and, at least in animal models, it modulates viral growth and suppresses antiviral responses. In 2016, the first evidence of PA-X expression in humans was the high titers of specific Abs to this protein found in sera from patients infected during the 2003 H7N7 outbreak occurred in The Netherlands (115). Moreover, the presence of Abs against the PB1-F2 protein were confirmed by immunoprecipitation and immunofluorescence assays in human convalescent sera and experimental infected mice (114).

**Figure 1:**
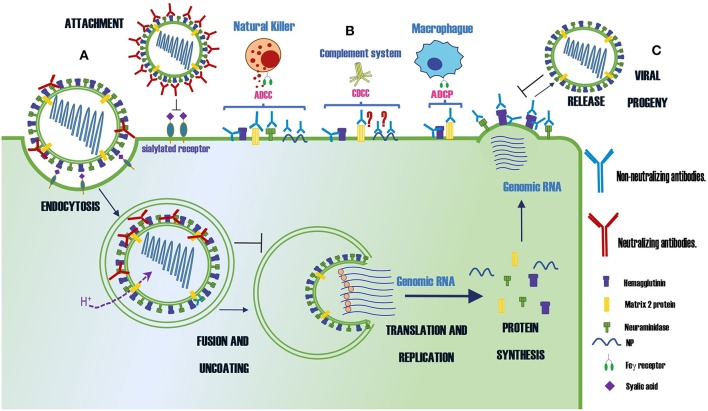
Summary of the protection mechanisms of neutralizing and non-neutralizing Abs specific for different proteins of the influenza virus. **(A)** Abs that neutralize the infection **(B)** Abs that control the infection by indirect mechanisms as ADCC, CDCC or ADCP **(C)** Abs that prevent the virus budding. The symbol ? indicates the mechanism of protection is controversial.

A summary of protective Abs against influenza virus and their mechanisms of protection is shown in Table 1 and Figure 1, respectively.

**Table 1:** Mechanisms of action of protective antibodies against influenza virus proteins in mouse and humans.

**Antibodies against**	**Mechanism**		**Confirmed with:**	
			**Mouse Abs**	**Human Abs**
HA (head)	Neutralizing (strain specific)	Block virus attachment to host cell	✓^a^	✓
	Broadly neutralizing		✓ S139/1^b^ (43)^c^	✓ CH65, C05, F045-092, F026-427, D1-8 (44–47)
	Non-neutralizing	ADCC		
		ADCP		
		CDCC		(92)
		Inhibit NA activity	✓ (30, 32)	
HA (stem)	Broadly neutralizing	Block fusion	✓ C179 (48)	✓ CR6261, CR8020, FI6, F10 (48–52)
	Non-neutralizing	ADCC		FI6 (84)
		ADCP	✓ (91)	✓ (91)
		CDCC		✓ (92)
		Inhibit NA activity	✓ (32)	✓ (31)
NA	Neutralizing	Not described		✓ (66)
	Non-neutralizing	NI-activity, interfere with viral release	✓ (76)	✓ (66)
		ADCC		✓ (87)
M2	Non-neutralizing	ADCC		✓ 1–10 (99)
		ADCP	✓ (98)	
		CDCC	✓ (101)	
NP	Non-neutralizing	ADCC		✓ (87)
		CDCC	✓ Low activity (123)	

a. *✓ indicates that the mechanism of action has been confirmed*.

b. *mAb name*.

c. *Reference in parenthesis*.

## Conclusion

Both neutralizing and non-neutralizing Abs can offer heterosubtypic protection against IAV. However, the Abs that recognize highly conserved epitopes are subdominant during the course of a natural infection or after vaccination. Therefore, efforts to build a universal vaccine with these antigenic determinants are being made, along with strategies for increasing their immunity. Nevertheless, despite the significant advances on the knowledge of heterosubtypic humoral immunity and the biology of B cells in animal models, further studies in humans are needed to define the viability of using them as a component of an anti-IAV universal vaccine or as a therapeutic measure.

## Author Contributions

HP-Q conceived this review and wrote the manuscript. FE-G directed the project and participated editing all the sections. DL-G created the figure and critically read the manuscript. LG-X critically read and edited the manuscript. All authors approved the final version of the manuscript. This work was done as a complementary academic activity of the Ph.D. program of HP-Q.

### Conflict of Interest Statement

The authors declare that the research was conducted in the absence of any commercial or financial relationships that could be construed as a potential conflict of interest.
